# Comprehensive Assessment of Biventricular and Biatrial Myocardial Strain Parameters at 4 Years Postpartum in a Cohort of Women with Previous Gestational Diabetes Mellitus

**DOI:** 10.3390/jcm14041271

**Published:** 2025-02-14

**Authors:** Andrea Sonaglioni, Federica Casieri, Gian Luigi Nicolosi, Stefano Bianchi, Michele Lombardo

**Affiliations:** 1 Division of Cardiology, IRCCS MultiMedica, 20123 Milan, Italy; michele.lombardo@multimedica.it; 2 Division of Gynecology and Obstetrics, IRCCS MultiMedica, 20123 Milan, Italy; federica.casieri@unimi.it (F.C.); stefano.bianchi@unimi.it (S.B.); 3 Division of Cardiology, Policlinico San Giorgio, 33170 Pordenone, Italy; gianluigi.nicolosi@gmail.com

**Keywords:** previous gestational diabetes mellitus, subclinical myocardial dysfunction, early carotid atherosclerosis, body mass index, glycosylated hemoglobin

## Abstract

**Background/Objectives**: No previous study has provided a comprehensive evaluation of all biventricular and biatrial myocardial strain parameters in women with previous gestational diabetes mellitus (pGDM). Accordingly, we aimed at investigating the structural and myocardial deformation properties of all cardiac chambers in a cohort of pGDM women at 4 years postpartum. **Methods**: A consecutive cohort of pGDM women was compared to a control group of healthy women with previous uncomplicated pregnancy, matched by age, ethnicity and gestational week, at 4 years postpartum. Both groups of women underwent transthoracic echocardiography (TTE) implemented with speckle-tracking echocardiography (STE) and subsequent carotid ultrasonography. The primary endpoint was subclinical myocardial dysfunction, defined as left-ventricular (LV) global longitudinal strain (GLS) < 20%, whereas the secondary endpoint was early carotid atherosclerosis, defined as common carotid artery (CCA) intima-media thickness (IMT) ≥ 0.7 mm. **Results**: A total of 32 pGDM women (39.1 ± 6.5 yrs) and 30 matched healthy controls (40.8 ± 5.0 yrs) were analyzed. Despite normal and similar systolic function on conventional TTE, all biventricular and biatrial strain parameters were significantly lower in pGDM women than controls. Mean follow-up period was 4.0 ± 1.9 yrs. During follow-up, 62.5% of pGDM women developed subclinical myocardial dysfunction, and 78.1% of them were diagnosed with early carotid atherosclerosis. Third-trimester BMI (OR 1.88, 95% CI 1.19–2.98) and third-trimester glycosylated hemoglobin (HbA1C) (OR 2.34, 95% CI 1.08–5.04) were independently associated with the primary endpoint. Third-trimester BMI and HbA1C also independently predicted the secondary endpoint. Third-trimester BMI > 27 kg/m^2^ and HbA1C > 33 mmol/mol showed the best sensitivity and specificity for predicting both endpoints. **Conclusions**: Women with a previous history of GDM complicated by overweight/obesity and uncontrolled diabetes have a significantly increased risk of subclinical myocardial dysfunction and early carotid atherosclerosis at 4 years postpartum.

## 1. Introduction

The global prevalence of gestational diabetes mellitus (GDM), defined as glucose intolerance with first onset during pregnancy, is approximately 15%, and prevalence rates are expected to continue rising [1]. Data from the literature indicate that GDM is associated with an increased risk of adverse maternal and neonatal outcomes [2,3,4]. Although GDM usually resolves after birth, recent evidence suggests that the impact persists over time. In this regard, women with a previous history of gestational diabetes mellitus (pGDM) were reported to be at risk of developing type 2 diabetes mellitus, metabolic syndrome and chronic kidney disease later in life [5,6,7,8]. The size of this risk association has been estimated to be a relative risk of almost 10 for type 2 diabetes mellitus and almost 2 for cardiovascular (CV) disease in the first decade postpartum [5,9,10]. The magnitude of these risks highlights the importance of intervening to prevent the onset of T2DM and the occurrence of adverse CV events, particularly in the early years after pregnancy [5]. For this reason, it is important to recognize the eventual abnormalities of cardiac structure and function in pGDM women at an early stage, prior the occurrence of adverse outcomes.

Transthoracic echocardiography (TTE) is the most widely used imaging technique for monitoring maternal cardiac function in pregnancy and postpartum. It precisely measures cardiac chamber cavity sizes, left-ventricular (LV) diastolic and systolic function and pulmonary hemodynamics [11]. Although the left-ventricular ejection fraction (LVEF) is the principal index of LV systolic function, it has several limitations, including the load dependence and the increased intra- and inter-rater variability [12,13]. Moreover, the LVEF does not provide information concerning regional wall motion [14]. For this reason, the LVEF can be normal during the initial stages of cardiac diseases, thus representing a late marker of LV systolic dysfunction [15].

Recent innovations in cardiac imaging have led to the introduction of speckle-tracking echocardiography (STE), which evaluates the myocardial deformation properties of cardiac chambers [16]. LV global longitudinal strain (GLS), assessed by STE analysis, can detect systolic dysfunction much earlier than the LVEF, thus identifying individuals with subclinical myocardial damage [17].

To date, several studies [18,19,20,21,22,23,24,25,26] have demonstrated that, despite a normal TTE-derived LVEF, LV-GLS was significantly reduced in GDM women compared to healthy pregnant women. Conversely, subclinical myocardial dysfunction has been poorly investigated in women with previous history of GDM (pGDM women) [27,28]. Given that the risk of CV events in pGDM women is higher within the first decade after pregnancy [9], we hypothesized that subclinical myocardial dysfunction might early involve all cardiac chambers in pGDM women during the first years postpartum. Accordingly, the present study aimed primarily at evaluating cardiac structure and function in a cohort of pGDM women in comparison to women with previous uncomplicated pregnancy at 4 years postpartum.

## 2. Materials and Methods

### 2.1. Patient Selection

The present study analyzed a consecutive series of pGDM women compared to a control group of normotensive healthy women with previous uncomplicated pregnancy, matched by age, ethnicity and gestational week, between February 2024 and April 2024. The two groups of women underwent delivery at our institution between March 2021 and June 2021.

Inclusion criteria were as follows: women with a previous history of GDM, defined according to the IADPSG criteria [29]. Exclusion criteria were as follows: previous evidence of any form of pregestational diabetes mellitus, concomitant cardiovascular, respiratory and/or renal diseases, gestational or pre-gestational hypertension, hemodynamic instability and poor acoustic windows.

The following data were collected by accessing women’s medical records available in the hospital archive: anagraphic age, ethnicity, body surface area, body mass index (BMI), waist-to-hip ratio (WHR), prevalence of main cardiovascular risk factors, parity, relevant comorbidities, blood pressure values, gestational week at diagnosis of GDM, delivery parameters, blood tests comprehensive of glycometabolic parameters and, finally, the third-trimester and current medical treatment.

Each woman included in the present study underwent anamnesis, physical examination, electrocardiogram (ECG), a TTE implemented with STE and, finally, a carotid ultrasonography.

The study protocol was approved by the Comitato Etico Territoriale Lombardia 5 (committee’s reference number 507/24, date of approval 22 October 2024).

### 2.2. Conventional Echocardiographic Examination

All echocardiograms were performed by using a Philips Sparq ultrasound machine (Philips, Andover, MA, USA) with a 2.5 MHz transducer.

The following conventional echocardiographic parameters were recorded: the aortic root and ascending aorta diameters; the relative wall thickness (RWT); the left-ventricular mass index (LVMi); the left-ventricular end-diastolic volume index (LVEDVi) and the left-ventricular end-systolic volume index (LVESVi); the left-ventricular ejection fraction (LVEF) as index of LV systolic function [30]; the left-atrial volume index (LAVi); the right-ventricular inflow tract (RVIT); and the tricuspid annular plane systolic excursion (TAPSE) as index of right-ventricular (RV) systolic function.

Doppler measurements included the E/A ratio and E/average e’ ratio as indices of LV diastolic function and left-ventricular filling pressure (LVFP), respectively [31]. Systolic pulmonary artery pressure (sPAP) was derived by the modified Bernoulli equation [32]. Finally, the TAPSE/sPAP ratio was measured as a noninvasive index of RV/pulmonary artery (PA) coupling [33].

### 2.3. Measurement of Epicardial Adipose Tissue Thickness

Epicardial adipose tissue (EAT) thickness was measured at the end of systole on the RV free wall from the echocardiographic parasternal long-axis view and was defined as a hypoechoic area adjacent to the right ventricle [34].

### 2.4. Hemodynamic Indices

The following hemodynamic parameters were obtained: systolic blood pressure (SBP), diastolic blood pressure (DBP) and mean arterial pressure (MAP), with the latter estimated as MAP = DBP + [(SBP − DBP/3)] [35], the stroke volume (SV), the cardiac output (CO), calculated as the product of the SV and heart rate [36], and the total peripheral resistance (TPR), calculated as TPR = MAP (kPa)/CO (L/min) × 80 [37].

To assess ventricular–arterial coupling (VAC), the following parameters were measured: (1) end-systolic pressure (ESP), estimated as 0.9 × brachial SBP [38]; (2) SV; (3) the effective arterial elastance index (EaI), calculated as EaI = ESP/SVindex ratio [39]; and (4) the end-systolic elastance index (EesI), estimated as EesI = ESP/(LVESVi − V0), assuming that V0 is negligible compared with LVESVi [39]. VAC was calculated as the EaI/EesI ratio.

### 2.5. Speckle-Tracking Echocardiography

STE analyses were performed offline on two-dimensional movies acquired from the three apical views (four-chamber, two-chamber and three-chamber) for LV longitudinal strain and from the three parasternal views (at the basal, mid and apical level) for LV circumferential strain using the Philips QLAB 10.3.1 ultrasound software [40].

A single bull’s-eye summary for LV-GLS and LV-global circumferential strain (GCS) was obtained, reporting both regional and global LV strain. The early peak diastolic strain rate was derived from longitudinal and circumferential measurements [40].

RV-GLS was measured from the apical four-chamber view by averaging regional strains obtained from the septal and lateral segments. Right-ventricular free-wall longitudinal strain (RV-FWLS) was calculated as the mean of the RV lateral basal, mid and apical segments without including septal segments [41].

Left-atrial (LA) strain was calculated by using a “biplane method”. The following measurements were performed: the peak positive LA longitudinal strain corresponding to left-atrial conduit strain (LAScd), the peak negative LA longitudinal strain corresponding to left-atrial contractile strain (LASct) and the sum of the two peaks corresponding to left-atrial reservoir strain (LASr). From the atrial strain measurements, the strain rate curves were derived [42]. Moreover, we calculated the LASr/E/average e’ ratio, an echocardiographic index of LA stiffness [43].

Right-atrial reservoir strain (RASr) was assessed from the apical four-chamber view by placing the markers on the edges of the tricuspid annulus and to the right-atrial roof.

Absolute values superior to 20% for LV-GLS [11], 23.3% for LV-GCS [44], 20% for RV-GLS [45], 39% for LASr [46] and 35% for RASr [47] were considered as the cut-off values of normality for comparison.

### 2.6. Carotid Ultrasonography

All carotid ultrasound examinations were performed by using the same Philips Sparq ultrasound machine with a 12 MHz transducer.

Based on a standardized protocol [48], average values of the intima-media thickness (IMT) and end-diastolic diameter (EDD) of the left and right common carotid arteries (CCAs) were measured in the distal CCA at 1 cm from the carotid bifurcation. Average carotid RWT was estimated as 2 × average IMT/average CCA-EDD, while the average CCA cross-sectional area (CSA) (mm^2^) was calculated as CCA-CSA = [π × (2 × average IMT + average CCA-EDD)/2)^2^ − π × (average CCA-EDD/2)^2^].

Considering that normal IMT values are age- and sex-dependent [49] and that the reference limit of IMT according to the age class of 40–49 yrs is 0.67 mm [50], a CCA-IMT ≥ 0.7 mm was the cut-off employed to define CCA intima-media thickening in our study population.

### 2.7. Statistical Analysis

The primary endpoint of this study was the occurrence of subclinical myocardial dysfunction, defined as an LV-GLS value < 20% in the presence of a preserved LVEF (≥55%) [11] in pGDM women at 4 years postpartum. The secondary endpoint was the occurrence of increased CCA-IMT (≥0.7 mm) [50] in the same cohort of pGDM women.

A sample size of 30 women with a previous history of GDM and 30 healthy controls reached 80% of statistical power to detect a two-point difference in the GLS magnitude (i.e., 20% vs. 18%) measured at 4 yrs postpartum in the two groups of women with a standard deviation of 2.5 for each parameter, using a two-sided equal-variance *t*-test with a level of significance (alpha) of 5%.

Given that all data were normally distributed, continuous variables were compared using a two-sample independent *t*-test, whereas categorical parameters were compared using the chi-squared test.

Logistic regression analyses were performed to identify the independent predictors of subclinical myocardial dysfunction and intima-media thickening in pGDM women at the 4-year follow-up. The following variables were included in the logistic regression analysis: third-trimester age (as a demographic parameter), third-trimester BMI (as an anthropometric variable), third-trimester glycosylated hemoglobin (as a glycometabolic index), third-trimester NLR (as a systemic marker of inflammation) and third-trimester MAP (as a hemodynamic index). For each variable investigated, correspondent odds ratios with 95% confidence intervals (CIs) were calculated.

ROC curve analysis was performed to establish the sensitivity and the specificity of the main statistically significant continuous variable for predicting both endpoints over the follow-up period. The area under the curve (AUC) was estimated.

Statistical analysis was performed with SPSS version 28 (SPSS Inc., Chicago, IL, USA).

## 3. Results

### 3.1. Clinical Findings

A total of 32 pGDM women and 30 age-, ethnicity- and gestational-week-matched healthy controls without any comorbidity were analyzed at 4 yrs postpartum.

The main clinical, obstetrical, hemodynamic and laboratory parameters collected in the two study groups at the third trimester of pregnancy are summarized in Table 1.

Compared to controls, the GDM women had a significantly higher third-trimester BMI (≥30 kg/m^2^ in 43.7% of women) and a significantly higher prevalence of dyslipidemia and family history of diabetes. BP measurements revealed significantly higher values in the GDM women than in controls, even in the absence of arterial hypertension (BP ≥ 140/90 mmHg). On third-trimester blood tests, the GDM women were found to have significantly higher inflammatory indices, serum total cholesterol and serum uric acid than controls. Overall, the GDM women showed good glycemic control. Approximately two-thirds of the GDM cases (68.7% of total) were diagnosed after 24 weeks of gestation, whereas one-third (31.3% of total) were diagnosed earlier in pregnancy. Among the GDM participants, 56.2% were on a diet, and the remaining 43.8% on insulin. The analysis of delivery parameters revealed that the GDM women underwent delivery much earlier than controls.

At the clinical visit performed at the 4-year follow-up, one-third of the pGDM women were affected by type 2 diabetes mellitus, dyslipidemia, arterial hypertension and obesity. Considering the average WHR obtained in the pGDM women (0.90 ± 0.16), the most prevalent obesity phenotype was the android one. Among the pGDM women, 12.5% of the total were treated with oral hypoglycemic agents and/or antihypertensive drugs (%), whereas only 6.2% of the total made regular use of statins (Table 2).

### 3.2. Instrumental Findings

Table 3 lists all the morphological, functional and hemodynamic parameters assessed by conventional TTE and carotid ultrasonography in the two groups of women at 4 yrs postpartum.

On TTE examination, biventricular and biatrial cavity sizes were similar in the two groups of women. Even in the absence of manifest pathological LV remodeling, the pGDM women were diagnosed with significantly greater RWT, LVMi and LA antero-posterior diameter than controls. LV systolic function, assessed by the LVEF, was normal and similar in both groups of women. Analysis of LV diastolic function revealed a significantly higher E/average e’ ratio in the pGDM women in comparison to controls. No significant valvulopathy was detected in both study groups. The assessment of pulmonary hemodynamics showed that both TAPSE and the TAPSE/sPAP ratio were significantly reduced in the women with previous GDM than in controls. Finally, EAT thickness was significantly increased in the pGDM women than in controls.

The analysis of the hemodynamic indices showed that SV and CO were significantly lower in the pGDM women than in controls, whereas the heart rate was similar in the two groups of women. In addition, TPRi was significantly increased in the pGDM women.

Concerning the VAC parameters, the pGDM women were found with significantly higher EaI than controls, while EesI was similar in the two groups of women; the resultant VAC (EaI/EesI ratio) was significant greater in the pGDM women than in controls.

On carotid ultrasonography, the average values of CCA-IMT, CCA-RWT and CCA-CSA were all significantly larger in the pGDM women than in controls.

Strain echocardiographic imaging revealed that most biventricular and biatrial myocardial strain and strain rate parameters were significantly reduced in the pGDM women in comparison to healthy controls. Overall, more than half of the pGDM women were diagnosed with lower biventricular and biatrial myocardial strain parameters in comparison to the accepted reference values [11,44,45,46,47]. Interestingly, approximately one-fifth of healthy controls were found with a mild attenuation of myocardial deformation indices (Table 4).

**Figure 1:**
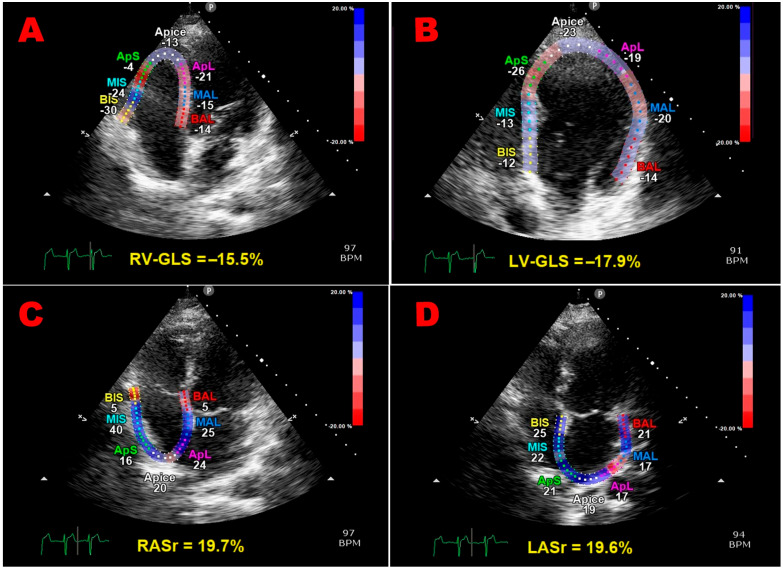
Representative examples of right ventricular (**A**), left ventricular (**B**), right atrial (**C**) and left atrial (**D**) longitudinal strain parameters measured from the apical four-chamber view in a pGDM woman included in the present study. All myocardial strain parameters were reduced in comparison to the accepted reference ranges. GLS, global longitudinal strain; LASr, left-atrial reservoir strain; LV, left-ventricular; pGDM, previous gestational diabetes mellitus; RASr, right-atrial reservoir strain; RV, right-ventricular.

The multi-panel image in Figure 1 illustrates examples of the biventricular and biatrial longitudinal strain parameters measured from the apical four-chamber view in a pGDM woman included in the present study.

### 3.3. Follow-Up Data

The mean follow-up period was 4.0 ± 1.9 yrs. During follow-up, no pGDM woman developed any symptoms or signs of cardiomyopathy. No major adverse CV event was recorded.

At the 4-year follow-up, approximately two-thirds of the pGDM women (62.5% of the total) developed subclinical myocardial dysfunction, as revealed by the STE analysis, and one-third of the total were diagnosed with type 2 diabetes mellitus (31.2% of the total) or arterial hypertension (31.2% of the total). Moreover, 25 pGDM women (78.1% of the total) were found with subclinical carotid atherosclerosis.

On logistic regression analysis, third-trimester BMI (OR 1.88, 95% CI 1.19–2.98, *p* = 0.03) and glycosylated hemoglobin (OR 2.34, 95% CI 1.08–5.04, *p* = 0.02) were independently associated with the primary endpoint (Table 5).

**Figure 2:**
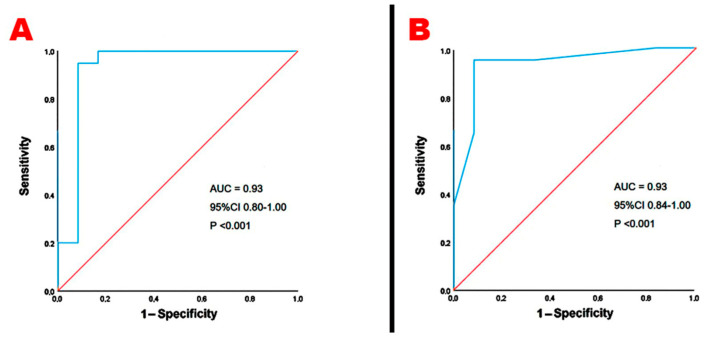
ROC curve analysis performed to establish the sensitivity and the specificity of third-trimester BMI (**A**) and third-trimester glycosylated hemoglobin (**B**) for predicting the primary endpoint. AUC, area under curve; BMI, body mass index; ROC, receiver operating characteristics.

ROC curve analysis revealed that both third-trimester BMI > 27 kg/m^2^ and third-trimester glycosylated hemoglobin > 33 mmol/mol had 95% sensitivity and 99% specificity for predicting GLS impairment in pGDM women at the 4-year follow-up (Figure 2A,B, respectively).

**Figure 3:**
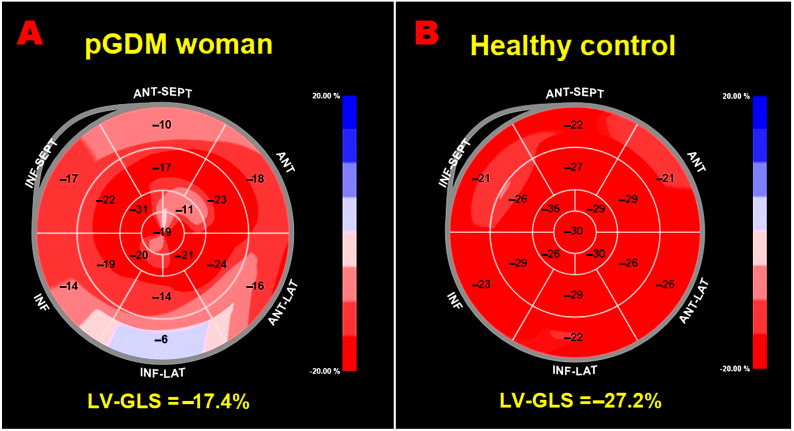
Examples of LV-GLS bull’s-eye plots obtained in a pGDM woman with previous pregnancy complicated by obesity and uncontrolled diabetes (**A**) and in a woman with previous uncomplicated pregnancy (**B**), respectively. GLS, global longitudinal strain; LV, left-ventricular; pGDM, previous gestational diabetes mellitus.

Figure 3 illustrates examples of GLS bull’s-eye plots obtained in a pGDM woman with third-trimester BMI > 27 kg/m^2^ and third-trimester glycosylated hemoglobin > 33 mmol/mol (A) and in a woman with previous uncomplicated pregnancy (B), respectively.

Third-trimester BMI (OR 1.35, 95% CI 1.02–1.79, *p* = 0.03) and third-trimester glycosylated hemoglobin (OR 1.37, 95% CI 1.00–1.88, *p* = 0.02) were also independently associated with increased CCA-IMT (≥0.7 mm) in pGDM women at 4 years postpartum (Table 6).

**Figure 4:**
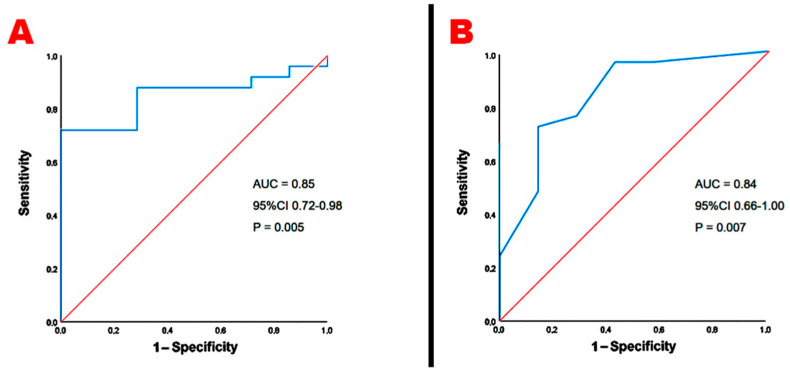
ROC curve analysis performed to establish the sensitivity and the specificity of third-trimester BMI (**A**) and third-trimester glycosylated hemoglobin (**B**) for predicting the secondary endpoint. AUC, area under curve; BMI, body mass index; ROC, receiver operating characteristics.

A third-trimester BMI > 27 kg/m^2^ and third-trimester glycosylated hemoglobin > 33 mmol/mol showed the best sensitivity and specificity for predicting the secondary endpoint (Figure 4A,B, respectively).

## 4. Discussion

### 4.1. Main Findings of the Present Study

This prospective case–control study demonstrated that compared to healthy women with previous uncomplicated pregnancy, pGDM women showed the following: (1) a subtle LV remodeling characterized by greater RWT and LVMi, with no evidence of LV concentric remodeling or LV concentric hypertrophy on TTE; (2) a moderate increase in the E/average e’ ratio, with no evidence of any pathological increase in LVFP; (3) a lower TAPSE and TAPSE/sPAP ratio without RV-PA uncoupling (defined as a TAPSE/sPAP ratio < 0.80) [26]; (4) a higher arterial elastance and VAC and (5) early carotid artery remodeling with a greater CCA-RWT and CCA-CSA. Despite a normal LVEF on conventional TTE, 2D-STE analysis highlighted a modest, but significant, impairment in almost all biventricular and biatrial myocardial strain parameters in the pGDM women vs. controls. No relevant CV event was recorded over the follow-up period. However, approximately two-thirds of the pGDM women showed subclinical myocardial dysfunction on strain echocardiographic imaging, and one-third of the total were found with type 2 diabetes mellitus or arterial hypertension at the 4-year follow-up. Third-trimester BMI and third-trimester glycosylated hemoglobin were the main prognostic indicators of both the primary and secondary endpoints. Notably, a third-trimester BMI > 27 kg/m^2^ and a third-trimester glycosylated hemoglobin > 33 mmol/mol were the best cut-off values for predicting subclinical myocardial dysfunction and subclinical atherosclerosis in pGDM women at 4 yrs postpartum.

### 4.2. Comparison with Previous Studies and Interpretation of Results

To the best of our knowledge, to date, only two studies [27,28] have evaluated myocardial strain parameters in pGDM women. In the CARDIA study [27], compared with women with non-GDM pregnancies, pGDM women had a significantly lower magnitude of LV-GLS, lateral e’ wave velocity and septal e’ wave velocity and a greater 20-year increase in LVMi. All these findings were independent of the subsequent development of type 2 diabetes mellitus. Aguilera J et al. [28] demonstrated that GDM women, evaluated at the third trimester of pregnancy, had a significantly higher LVMi and E/average e’ ratio and a significantly lower E/A and LV-GLS than healthy controls; the impairment in both the E/A ratio and LV-GLS proved to be persistent at 6 months after delivery in the pGDM group.

Differently from the above-mentioned studies, the present study evaluated not only LV-GLS but also performed a comprehensive assessment of the myocardial deformation properties of all cardiac chambers in the pGDM women included. The subclinical myocardial dysfunction involved both ventricles and atria. To the best of our knowledge, this is the first study that has evaluated the structure and function of all cardiac chambers in pGDM women in the first decade postpartum. The results of the present study confirmed the superiority of strain echocardiographic imaging over conventional TTE in detecting a subclinical impairment in myocardial deformation indices in the absence of any signs and symptoms of cardiomyopathy and in the presence of a preserved LVEF (≥55%).

According to the most recent data in the literature, the occurrence of both subclinical myocardial dysfunction and carotid artery remodeling in pGDM women would exist even in the absence of type 2 diabetes [51,52,53]. It is known that obesity [54,55], dyslipidemia [56], arterial hypertension [57], insulin resistance and hyperinsulinemia [58] are all risk factors for the early deterioration of myocardial strain parameters assessed by STE. In light of our findings, even a brief period of exposure to the hemodynamic overload related to overweight/obesity together with hyperinsulinemia due to uncontrolled GDM might have contributed to the persistent impairment in the biventricular and biatrial myocardial strain indices observed in the pGDM women.

Given that obesity [59] and hyperinsulinemia [60] have been strongly correlated with the occurrence of heart failure with a preserved ejection fraction, our results would suggest that pGDM women with obesity and uncontrolled diabetes might have an increased risk of developing this echocardiographic phenotype of heart failure.

Consistent with previous studies [61,62,63,64], the present study also confirmed the strong association between a previous history of GDM and the subsequent occurrence of subclinical carotid atherosclerosis. This association is triggered by third-trimester overweight/obesity and increased glycosylated hemoglobin and is accelerated by age, smoking, dyslipidemia and arterial hypertension, which may all synergically contribute to early carotid atherosclerosis [65].

The increase in VAC and the concomitant decrease in RV/PA coupling observed in our cohort of pGDM women was attributed by our study group to an increased stiffening involving both systemic and pulmonary circulation. It is likely that an increased LV and RV afterload might have favored the attenuation of biventricular deformation properties, similarly to what was observed in large cohorts of hypertensive individuals [66].

The attenuation of myocardial deformation indices detected in more than half of the pGDM women and in approximately one-fifth of healthy controls may also be explained by the potential influence exerted by anthropometrics, such as pectus excavatum, abdominal and/or thoracic adiposity, on the biventricular and biatrial mechanics [67,68]. Indeed, it is not possible to exclude that a “cardiac restriction” due to a narrow antero-posterior thoracic diameter and/or compressive phenomena may have contributed to the reduced myocardial deformation indices, particularly at a basal level, in some pGDM women and/or healthy controls. However, this methodological issue was not investigated in the present study.

### 4.3. Implications for Clinical Practice

The results of the present study revealed that cardiovascular functional changes that begin during the third trimester of pregnancy may persist long after delivery and may contribute to the future occurrence of subclinical myocardial dysfunction and early carotid atherosclerosis within the first decade postpartum. Considering the increased CV risk associated with GDM diagnosis, the American guidelines [69,70] recommend an OGTT at 4 to 12 weeks postpartum and then a repeat screening, comprehensive of fasting plasma glucose and glycosylated hemoglobin every 1 to 3 years, for all women with a previous history of GDM. Weight loss programs and adequate glycemic control should be initiated earlier in pregnancy and continued in the postpartum period for all GDM women with overweight/obesity. In this regard, recent evidence indicates the beneficial effect of physical activity before pregnancy for reducing the risk of GDM and CV complications in pregnancy [71]. In addition, modifiable CV risk factors, including hypertension, visceral adiposity and dyslipidemia, should be corrected in order to reduce the CV risk in such women. Due to their protective pleiotropic effects [72], statins and anti-hypertensive agents should be considered in pGDM women with concomitant arterial hypertension and dyslipidemia. In light of our findings, strain echocardiographic imaging should be considered for implementation in clinical practice, particularly during pregnancies complicated by GDM and during the first decade postpartum. This innovative methodology may detect subtle changes in myocardial deformation indices, providing incremental diagnostic and prognostic information on biventricular and biatrial mechanics over conventional TTE examination.

### 4.4. Limitations of the Study

The main study limitations were its monocentric nature and the limited number of pGDM women analyzed. However, the number of pGDM women included was justified by an accurate sample size calculation. Moreover, our study group was evaluated by TTE implemented with STE at only 4 years postpartum; therefore, it is not possible to establish whether the impairment in biventricular and biatrial deformation was already present during the previous pregnancy complicated by GDM. In addition, biventricular and biatrial myocardial deformation indices were obtained by using the same software employed for LV-GLS assessment, the only one available at our institution. It is noteworthy that strain echocardiographic imaging is strongly dependent on good image quality, on frame rates, on the operator’s experience, on the loading conditions, on the ultrasound system used for the analysis and on the chest wall conformation [73,74,75,76]. Finally, the pGDM women included in the present study did not perform blood tests comprehensive of C-reactive protein, N-terminal pro-B-type natriuretic peptide (NT-proBNP) and Homeostatic Model Assessment for Insulin Resistance (HOMA-IR), which were not foreseen in the research protocol. These inflammatory, hemodynamic and metabolic markers would have contributed to better understanding the pathophysiological mechanisms of both cardiac and carotid artery remodeling detected in pGDM women at 4 years postpartum. Future research should incorporate intermediate evaluations to better track CV changes from late gestation through the 4-year postpartum period [77].

## 5. Conclusions

Compared to women with previous uncomplicated pregnancy, women with a previous history of GDM complicated by overweight/obesity and uncontrolled diabetes have a significantly increased risk of subclinical myocardial dysfunction and early carotid atherosclerosis at 4 years postpartum.

Strain echocardiographic imaging may allow for the early identifying of, among pGDM women, those with subclinical myocardial dysfunction who might benefit from a closer clinical follow-up and/or a more aggressive medical treatment aimed at reducing the risk of CV complications later in life.

## Figures and Tables

**Table 1:** Clinical, obstetrical, hemodynamic and laboratory parameters collected in GDM women and controls at the third trimester of pregnancy. Data are expressed as mean ± SD or as number (percentage). Significant *p*-values are in bold. BMI, body mass index; BSA, body surface area; DBP, diastolic blood pressure; eGFR, estimated glomerular filtration rate; GDM, gestational diabetes mellitus; HR, heart rate; MAP, mean arterial pressure; NLR, neutrophil-to-lymphocyte ratio; PPH, postpartum hemorrhage; PROM, premature rupture of membranes; RDW, red blood cells distribution width; SBP, systolic blood pressure.

	GDM Women (*n* = 32)	Controls (*n* = 30)	*p*-Value
**Demographics, anthropometrics and obstetrics**			
Age (yrs)	34.1 ± 6.5	35.8 ± 5.0	0.26
Caucasian ethnicity (%)	16 (50.0)	19 (63.3)	0.29
Third-trimester BSA (m^2^)	1.86 ± 0.19	1.77 ± 0.15	**0.04**
Third-trimester BMI (kg/m^2^)	29.5 ± 6.0	26.6 ± 3.8	**0.03**
Obesity (BMI ≥ 30 kg/m^2^) (%)	14 (43.7)	5 (16.7)	**0.02**
Pluriparous (%)	16 (50.0)	13 (43.3)	0.59
Gestational age (weeks)	36.2 ± 1.8	36.6 ± 1.5	0.35
**Cardiovascular risk factors**			
Smoking (%)	5 (15.6)	6 (20.0)	0.65
Dyslipidemia (%)	16 (50.0)	5 (16.7)	**0.005**
Family history of diabetes (%)	16 (50.0)	3 (10.0)	**<0.001**
**Hemodynamics**			
HR (bpm)	86.8 ± 14.9	88.3 ± 8.8	0.63
SBP (mmHg)	108.8 ± 11.3	92.5 ± 8.6	**<0.001**
DBP (mmHg)	68.0 ± 7.0	59.3 ± 4.5	**<0.001**
MAP (mmHg)	81.6 ± 7.1	70.4 ± 5.5	**<0.001**
**Third-trimester blood tests and glycometabolic parameters**			
Serum hemoglobin (g/dL)	11.7 ± 1.1	11.3 ± 1.5	0.23
RDW (%)	15.3 ± 2.4	13.8 ± 2.1	**0.01**
NLR	4.4 ± 1.7	2.1 ± 0.5	**<0.001**
eGFR (mL/min/m^2^)	128.3 ± 12.6	133.6 ± 28.9	0.35
Serum total cholesterol (mg/dL)	253.75 ± 36.6	171.0 ± 11.2	**<0.001**
Serum uric acid (mg/dL)	4.9 ± 1.1	4.2 ± 0.6	**0.003**
Gestational age at diagnosis of GDM (weeks)	24.0 ± 5.8	/	/
Glycosylated hemoglobin (mmol/mol)	34.7 ± 4.1	/	/
**Antidiabetic treatment**			
Diet (%)	18 (56.2)	/	/
Insulin (%)	14 (43.8)	/	/
**Delivery parameters**			
Gestational week at delivery (weeks)	38.4 ± 0.9	39.1 ± 1.4	**0.02**
PROM (%)	3 (9.4)	1 (3.3)	0.33
Cesarean delivery (%)	8 (25.0)	10 (33.3)	0.47
PPH (%)	2 (6.2)	3 (10.0)	0.59
Neonatal birth weight (g)	3361.2 ± 292.6	3381.2 ± 480.5	0.84

**Table 2:** Clinical characteristics of the two study groups at 4 years postpartum. Data are expressed as mean ± SD or as number (percentage). E Significant *p*-values are in bold. BMI, body mass index; BP, blood pressure; BSA, body surface area; DBP, diastolic blood pressure; MAP, mean arterial pressure; pGDM, previous gestational diabetes mellitus; SBP, systolic blood pressure; WHR, waist-to-hip ratio.

	pGDM Women (*n* = 32)	Controls (*n* = 30)	*p*-Value
**Demographics and anthropometrics**			
Age (yrs)	39.1 ± 6.5	40.8 ± 5.0	0.26
Caucasian ethnicity (%)	16 (50.0)	19 (63.3)	0.29
BSA (m^2^)	1.76 ± 0.17	1.66 ± 0.14	**0.01**
BMI (kg/m^2^)	27.9 ± 4.5	22.2 ± 2.8	**<0.001**
Obesity (BMI ≥ 30 kg/m^2^) (%)	11 (34.4)	3 (10.0)	**0.02**
WHR	0.90 ± 0.16	0.78 ± 0.15	**0.003**
**Cardiovascular risk factors**			
Smoking (%)	5 (15.6)	6 (20.0)	0.65
Type 2 diabetes mellitus (%)	10 (31.2)	1 (3.3)	**0.004**
Dyslipidemia (%)	10 (31.2)	2 (6.7)	**0.01**
**Blood pressure parameters**			
SBP (mmHg)	122.4 ± 13.2	113.2 ± 11.1	**0.004**
DBP (mmHg)	76.2 ± 9.1	70.4 ± 9.4	**0.02**
MAP (mmHg)	91.6 ± 9.6	84.6 ± 8.9	**0.04**
BP ≥ 140/90 mmHg at clinical visit (%)	10 (31.2)	2 (6.7)	**0.01**
**Comorbidities**			
Hypothyroidism (%)	3 (9.4)	8 (26.7)	0.07
**Current medical treatment**			
Oral hypoglycemic agents (%)	4 (12.5)	1 (3.3)	0.18
Antihypertensive drugs (%)	4 (12.5)	1 (3.3)	0.18
Statins (%)	2 (6.2)	1 (3.3)	0.59

**Table 3:** Morphological, functional and hemodynamic parameters assessed by conventional transthoracxic echocardiography and carotid ultrasonography in the two groups of women at 4 years postpartum. Data are expressed as mean ± SD or as number (percentage). Significant *p*-values are in bold. A-P, antero-posterior; CCA, common carotid artery; COi, cardiac output index; CSA, cross-sectional area; EAT, epicardial adipose tissue; EaI, arterial elastance index; EDD, end-diastolic diameter; EesI, end-systolic elastance index; ESP, end-systolic pressure; IMT, intima-media thickness; IVC, inferior vena cava; IVS, interventricular septum; LA, left-atrial; LAVi, left-atrial volume index; LV, left-ventricular; LV-EDD, left-ventricular end-diastolic diameter; LVEDVi, left-ventricular end-diastolic volume index; LVESVi, left-ventricular end-systolic volume index; MR, mitral regurgitation; pGDM, previous gestational diabetes mellitus; PW, posterior wall; RVIT, right-ventricular inflow tract; RWT, relative wall thickness; sPAP, systolic pulmonary artery pressure; SVi, stroke volume index; TAPSE, tricuspid annular plane systolic excursion; TPRi, total peripheral resistance index; TR, tricuspid regurgitation.

	pGDM Women (*n* = 32)	Controls (*n* = 30)	*p*-Value
**Yrs postpartum**	4.0 ± 1.9	4.1 ± 2.1	0.84
**Conventional echoDoppler parameters**			
IVS (mm)	9.3 ± 1.8	7.6 ± 1.2	**<0.001**
LV-PW (mm)	7.6 ± 0.9	6.6 ± 1.0	**<0.001**
LV-EDD (mm)	44.0 ± 3.4	44.4 ± 2.7	0.61
RWT	0.34 ± 0.05	0.30 ± 0.05	**0.003**
LVMi (g/m^2^)	66.7 ± 10.9	57.7 ± 9.7	**0.001**
Normal LV geometric pattern (%)	29 (90.6)	29 (96.7)	0.33
LV concentric remodeling (%)	3 (9.4)	1 (3.3)	0.33
LVEDVi (mL/m^2^)	34.9 ± 6.15	35.3 ± 5.6	0.79
LVESVi (mL/m^2^)	11.8 ± 2.6	11.9 ± 2.5	0.88
LVEF (%)	65.8 ± 3.7	65.9 ± 4.8	0.93
E/A ratio	1.24 ± 0.31	1.34 ± 0.31	0.21
E/average e’ ratio	9.25 ± 3.01	5.14 ± 1.34	**<0.001**
LA A-P diameter (mm)	36.2 ± 3.3	33.6 ± 4.1	**0.008**
LAVi (mL/m^2^)	29.0 ± 7.3	27.4 ± 7.3	0.39
Mild MR (*n*, %)	7 (21.9)	9 (30.0)	0.46
Mild TR (*n*, %)	8 (25)	10 (33.3)	0.47
RVIT (mm)	29.7 ± 2.6	29.5 ± 3.0	0.78
TAPSE (mm)	23.9 ± 3.7	26.4 ± 3.6	**0.009**
IVC (mm)	16.6 ± 3.6	17.0 ± 3.9	0.68
sPAP (mmHg)	25.0 ± 4.9	22.8 ± 2.2	**0.03**
TAPSE/sPAP ratio	0.97 ± 0.19	1.17 ± 0.18	**<0.001**
Aortic root (mm)	29.0 ± 3.4	29.1 ± 2.6	0.89
Ascending aorta (mm)	28.9 ± 3.4	28.8 ± 3.1	0.90
End-systolic EAT (mm)	6.7 ± 1.3	4.1 ± 1.4	**<0.001**
**Hemodynamic indices**			
HR (bpm)	77.6 ± 11.1	75.5 ± 11.9	0.46
ESP (mmHg)	110.2 ± 11.9	101.9 ± 10.0	**0.004**
SVi (mL/m^2^)	32.2 ± 6.1	39.4 ± 9.1	**<0.001**
COi (L/min/m^2^)	2.5 ± 0.4	2.9 ± 0.7	**0.007**
TPRi (dyne.sec/cm^5^)/m^2^	3060.7 ± 669.6	2427.5 ± 620.6	**<0.001**
EaI (mmHg/mL/m^2^)	1.24 ± 0.48	1.00 ± 0.26	**0.02**
EesI (mmHg/mL/m^2^)	3.25 ± 1.07	3.28 ± 0.89	0.91
EaI/EesI ratio	0.39 ± 0.10	0.31 ± 0.09	**0.001**
**Carotid parameters**			
Av. CCA-EDD (mm)	6.76 ± 0.46	6.64 ± 0.44	0.29
Av. CCA-IMT (mm)	0.91 ± 0.26	0.62 ± 0.19	**<0.001**
Av. CCA-IMT ≥ 0.7 mm (%)	25 (78.1)	7 (23.3)	**<0.001**
Av. CCA-RWT	0.27 ± 0.08	0.19 ± 0.06	**<0.001**
Av. CCA-CSA (mm^2^)	22.0 ± 7.4	14.2 ± 4.9	**<0.001**

**Table 4:** Biventricular and biatrial strain parameters measured by speckle-tracking echocardiography in the two study groups at 4 years postpartum. Data are expressed as mean ± SD or as a number (percentage). Significant *p*-values are in bold. FWLS, free-wall longitudinal strain; GCS, global circumferential strain; GCSR, global circumferential strain rate; GLS, global longitudinal strain; GLSR, global longitudinal strain rate; GSRE, global early-diastolic strain rate; GSRL, global late-diastolic strain rate; LAScd, left-atrial conduit strain; LASct, left-atrial contractile strain; LASr, left-atrial reservoir strain; LV, left-ventricular; pGDM, previous gestational diabetes mellitus; RAScd, right-atrial conduit strain; RASct, right-atrial contractile strain; RASr, right-atrial reservoir strain; RV, right-ventricular.

STE VARIABLES	pGDM Women (*n* = 32)	Controls (*n* = 30)	*p*-Value
LV-GLS (%)	19.5 ± 2.6	22.3 ± 2.3	**<0.001**
LV-GLSR (s^−1^)	1.1 ± 0.1	1.2 ± 0.1	**<0.001**
LV-GCS (%)	22.8 ± 4.48	26.7 ± 4.4	**0.001**
LV-GCSR (s^−1^)	1.6 ± 0.3	1.7 ± 0.2	0.13
LAScd (%)	29.8 ± 8.9	36.3 ± 7.7	**0.003**
LASct (%)	7.3 ± 4.2	9.5 ± 4.1	**0.04**
LASr (%)	37.1 ± 9.2	45.7 ± 8.0	**<0.001**
LASr/E/e’	4.4 ± 1.8	9.5 ± 3.2	**<0.001**
LA-GSR (s^−1^)	1.9 ± 0.5	2.3 ± 0.5	**0.002**
LA-GSRE (s^−1^)	2.4 ± 0.7	3.1 ± 0.8	**<0.001**
LA-GSRL (s^−1^)	2.5 ± 0.6	2.8 ± 0.5	**0.04**
RV-FWLS (%)	19.9 ± 3.8	22.0 ± 3.5	**0.03**
RV-GLS (%)	18.8 ± 3.9	20.9 ± 3.4	**0.03**
RV-GLSR (s^−1^)	1.1 ± 0.2	1.3 ± 0.2	**<0.001**
RAScd (%)	26.3 ± 11.7	34.6 ± 10.1	**0.004**
RASct (%)	6.1 ± 4.46	7.5 ± 5.4	0.27
RASr (%)	32.4 ± 11.0	42.1 ± 9.9	**<0.001**
RA-GSR (s^−1^)	2.0 ± 0.9	2.5 ± 0.6	**0.01**
RA-GSRE (s^−1^)	1.9 ± 0.6	2.3 ± 0.7	**0.02**
RA-GSRL (s^−1^)	2.0 ± 0.6	2.5 ± 0.8	**0.007**
**PERCENTAGE OF WOMEN WITH IMPAIRED STE PARAMETERS IN COMPARISON TO THE ACCEPTED NORMAL VALUES**			
LV-GLS < 20% (%)	20 (62.5)	4 (13.3)	**<0.001**
LV-GCS < 23.3% (%)	16 (50.0)	7 (23.3)	**0.03**
LASr < 39% (%)	18 (56.3)	5 (16.7)	**0.001**
RV-GLS < 20% (%)	19 (59.4)	7 (23.3)	**0.004**
RASr < 35% (%)	20 (62.5)	8 (26.7)	**0.005**

**Table 5:** Univariate and multivariate logistic regression analyses performed for identifying the independent predictors of subclinical myocardial dysfunction in pGDM women at 4 yrs postpartum. BMI, body mass index; MAP, mean arterial pressure; NLR, neutrophil-to-lymphocyte ratio; pGDM, previous gestational diabetes mellitus.

	UNIVARIATE LOGISTIC REGRESSION ANALYSIS			MULTIVARIATE LOGISTIC REGRESSION ANALYSIS		
VARIABLES	OR	95% CI	*p*-Value	OR	95% CI	*p*-Value
Third-trimester age (yrs)	1.08	0.96–1.21	0.21			
Third-trimester BMI (kg/m^2^)	1.87	1.24–2.83	**0.003**	1.88	1.19.2.98	**0.03**
Third-trimester glycosylated hemoglobin (mmol/mol)	2.30	1.35–3.94	**0.002**	2.34	1.08–5.04	**0.02**
Third-trimester NLR	1.89	1.08–3.33	**0.03**	1.69	0.64–4.45	0.28
Third-trimester MAP	1.01	0.94–1.09	0.77			

**Table 6:** Univariate and multivariate logistic regression analyses performed for identifying the independent predictors of early carotid atherosclerosis in pGDM women at 4 yrs postpartum. BMI, body mass index; MAP, mean arterial pressure; NLR, neutrophil-to-lymphocyte ratio; pGDM, previous gestational diabetes mellitus.

	UNIVARIATE LOGISTIC REGRESSION ANALYSIS			MULTIVARIATE LOGISTIC REGRESSION ANALYSIS		
VARIABLES	OR	95% CI	*p*-Value	OR	95% CI	*p*-Value
Third-trimester age (yrs)	1.37	1.09–1.70	**0.005**	1.06	0.94–1.19	0.32
Third-trimester BMI (kg/m^2^)	1.40	1.09–1.82	**0.01**	1.35	1.02–1.79	**0.03**
Third-trimester glycosylated hemoglobin (mmol/mol)	1.37	1.08–1.74	**0.009**	1.37	1.00–1.88	**0.02**
Third-trimester NLR	1.34	0.81–2.22	0.25			
Third-trimester MAP	1.06	0.95–1.17	0.29			

## Data Availability

Data extracted from included studies will be publicly available on Zenodo (https://zenodo.org, accessed on 31 October 2024).
